# Development and Exploratory Validation of the Assisting Mealtime Scale for Dementia Care: Nursing Staff Perspectives on Mealtime Support

**DOI:** 10.1111/nhs.70343

**Published:** 2026-05-06

**Authors:** Hansen Tang, WenPeng You, Kazem Razaghi, Fung Kuen (Tebbin) Koo, Hui‐Chen (Rita) Chang

**Affiliations:** ^1^ School of Nursing and Midwifery, Parramatta South Campus Western Sydney University Sydney Australia; ^2^ Adelaide Medical School The University of Adelaide Adelaide Australia; ^3^ Adelaide Nursing School The University of Adelaide Adelaide Australia; ^4^ Sydney Nursing School, Faculty of Medicine & Health The university of Sydney Sydney New South Wales Australia

**Keywords:** eating challenge, factor analysis, people with dementia, reliability, targeted training, validity

## Abstract

Mealtime assistance for people living with dementia presents care‐related challenges, yet few validated instruments assess nursing staff perspectives. This study developed and conducted an exploratory validation of the Assisting Mealtime Scale. A multi‐phase instrument development process included literature review, expert consultation, content validation, pilot testing, and forward–backward translation. The 19‐item scale was administered to nursing staff in aged care settings in Australia and China (*N* = 240). Exploratory factor analysis using principal axis factoring with Promax rotation examined factor structure. Sampling adequacy was assessed using the Kaiser–Meyer–Olkin test and Bartlett's test of sphericity. Internal consistency was evaluated using Cronbach's alpha. Sampling adequacy was excellent (KMO = 0.891). A three‐factor solution explained 55.25% of total variance. Factors represented Perceived Knowledge and Skill Gaps, Time and Task Pressure, and Attitudes Toward mealtime assistance and Autonomy. The overall scale demonstrated good reliability (*α* = 0.890). The scale demonstrates preliminary construct validity and reliability. Further confirmatory and cross‐cultural validation is required.

## Key Points

- Supporting people living with dementia during mealtimes involves complex clinical, emotional, and organizational challenges for nursing staff.
- The Assisting Mealtime Scale was developed as an exploratory instrument to assess nursing staff perspectives on dementia‐related mealtime care, providing preliminary evidence of its structure and internal consistency.
- Three domains were identified: Perceived Knowledge and Skill Gaps, Time and Task Pressure, and Attitudes Toward Mealtime Assistance and Autonomy.
- The scale may assist aged care services in identifying perceived barriers, training needs, and organizational constraints to inform targeted staff education and workforce support initiatives.
- Further psychometric validation using confirmatory factor analysis and cross‐cultural measurement testing is required before broader clinical or research application.

## 1 Introduction

The global population is aging rapidly, leading to a substantial increase in the number of older adults living with dementia (LoBuono and Milovich Jr 2023). It is estimated that more than 50 million people worldwide currently live with dementia, and this number is projected to rise to 82 million by 2030 and 152 million by 2050 (WHO 2023). Dementia is a progressive condition characterized by impairments in memory, cognition, and functional abilities, which increasingly affect daily activities and quality of life as the disease advances (Rotenberg and Dawson 2022).

As dementia progresses, individuals commonly experience changes in eating behavior and increasing difficulty with food intake (Cipriani 2016, Chang et al. 2017). Eating behavior in older adults is influenced by a complex interplay of physiological, pathological, and psychological factors, making nutritional care highly individualized (Cipriani 2016). In the early stages of dementia, changes in eating behavior may be subtle and require minimal assistance. However, in moderate to advanced stages, individuals often experience more pronounced difficulties, including reduced appetite, food refusal, difficulty grasping utensils, impaired chewing, and swallowing problems (Passos et al. 2024). In severe cases, dysphagia and aspiration can occur, posing serious health risks and increasing morbidity and mortality (Figueredo 2023; Boateng et al. 2018).

Eating difficulties among people with dementia are associated with inadequate food and fluid intake, contributing to dehydration, unintended weight loss, and malnutrition. Difficulty initiating eating is a particularly significant risk factor, especially among those residing in aged care settings (Cipriani 2016). Evidence suggests that approximately 20% of aged care setting residents are malnourished, while up to 56% are at risk of malnutrition (Shune and Linville 2019; Bell et al. 2015). These nutritional challenges not only compromise physical health but also negatively affect functional status, recovery, and overall well‐being.

Importantly, not all factors contributing to eating difficulties in people with dementia are directly attributable to cognitive decline (Fostinelli et al. 2020). Coexisting conditions such as depression, fatigue, medication side effects (including antipsychotic use), and reduced motivation can further exacerbate eating difficulties (Pilgrim et al. 2015; Calsolaro et al. 2021). Addressing these multifactorial challenges requires comprehensive and responsive care approaches that extend beyond individual symptoms to encompass care practices and environmental factors (Chang et al. 2026).

Nursing staff play a central role in supporting people with dementia during mealtimes, particularly in aged care settings where they are the primary caregivers. Effective nursing interventions, including personalized dietary planning, supportive feeding techniques, and strategies that address psychological and physiological contributors, have been shown to improve food intake, nutritional status, and hydration among people with dementia (LoBuono and Milovich Jr 2023; Figueredo 2023; Passos et al. 2024). However, despite the recognized importance of nursing involvement, the factors influencing how nursing staff support eating during mealtimes are not always well understood or systematically assessed. These factors may include perceived knowledge and skill gaps, emotional strain, time pressures, organizational constraints, and challenges in balancing autonomy with safety during meals.

Although several instruments are available to assess eating behaviors, eating difficulties, and dining environments for people living with dementia, few tools are designed to evaluate the perspectives and experiences of nursing staff who provide mealtime assistance. Existing measures primarily focus on resident behaviors or environmental factors and therefore overlook the perceived knowledge gaps, emotional demands, and organizational constraints that shape how mealtime care is delivered in practice (Whear et al. 2014). For example, the Edinburgh Feeding Assessment Tool is widely used to assess feeding difficulties in people with dementia; however, it centers on resident behaviors and does not capture the skills, perceived challenges, or contextual pressures experienced by nursing staff during mealtime care (Uyar et al. 2022). Given that nursing staff perceptions of preparedness, task difficulty, emotional strain, and organizational barriers influence how mealtime support is delivered and sustained, the absence of staff‐focused assessment tools represents an important gap in dementia care measurement. The Assisting Mealtime Scale was therefore developed to address this gap by capturing nursing staff perceptions, emotional strain, and organizational challenges associated with dementia‐related mealtime care (Jung 2022).

Assessing nursing staff perspectives on dementia‐related mealtime care is essential for identifying perceived barriers, training needs, and areas requiring organizational support (Faraday et al. 2019; Li et al. 2021b). A dedicated instrument could provide structured insights into staff experiences and contextual constraints, thereby informing targeted education, workforce development strategies, and quality improvement initiatives in aged care settings (Jung 2022). Such an approach has the potential to enhance both care practices and nutritional outcomes for people living with dementia. Therefore, the aim of this study was to develop and conduct an initial exploratory validation of a survey instrument designed to assess nursing staff perspectives and experiences in supporting people living with dementia during mealtimes. Using exploratory factor analysis (EFA), this study aimed to examine the underlying structure of the Assisting Mealtime Scale and to provide preliminary evidence of its psychometric properties. As an initial, exploratory assessment tool, the scale may offer potential to inform future intervention development and support improvements in mealtime care practices. However, further validation is required to confirm its robustness and broader applicability in aged care settings.

## 2 Methods

### 2.1 Study Design and Ethical Approval

The development and exploratory validation of the Assisting Mealtime Scale followed a structured, multi‐phase process in accordance with established guidelines for instrument development (372013). The process comprised three sequential phases: (1) item generation and domain identification; (2) content validation and preliminary refinement; and (3) exploratory psychometric evaluation. Each phase informed subsequent stages, ensuring a systematic and iterative approach to scale development.

Ethical approval for the study was obtained from the University Human Research Ethics Committee (HREC approval number: H15406).

### 2.2 Phase 1: Item Development

#### 2.2.1 Identification of Domains and Item Generation

A comprehensive literature review was conducted using PubMed, CINAHL, and Scopus, covering publications from database inception to February 2024. The search focused on mealtime challenges among people living with dementia, nursing staff behaviors and experiences, and existing measurement tools related to mealtime assistance and nutritional care. Key themes identified across the literature included emotional responses to eating difficulties, confidence in managing complex behaviors, communication challenges, ethical tensions, perceived knowledge gaps, and workflow‐related pressures.

To ensure that the scale reflected frontline nursing staff perspectives, the research team engaged in iterative discussions integrating empirical evidence with professional expertise in dementia care. Based on this process, an initial pool of approximately 23 items was generated. These items were provisionally organized into five preliminary domains: Knowledge and Skill Deficit, Care Philosophy and Approach, Intervention Preference, Helplessness and Pessimism, and Workload and Time Management. These domains were conceptual guides for item development rather than fixed structural assumptions and were subject to empirical refinement during subsequent psychometric evaluation.

All items were formulated as declarative statements and rated using a conventional 5‐point Likert scale (1 = *strongly disagree* to 5 = *strongly agree*). Five‐point Likert scales are widely recognized as appropriate for assessing attitudinal and perceptual constructs and support nuanced response patterns while maintaining usability (Sullivan and Artino Jr. 2013; Rotenberg and Dawson 2022). A neutral midpoint (“neither agree nor disagree”) was retained to allow balanced responses (Rush et al. 2017). Reverse‐worded items were not included, as reverse coding may introduce measurement error and increase cognitive burden in survey instruments (Saucedo Figueredo et al. 2023).

#### 2.2.2 Content Validity

Content validity was established using a structured, multi‐stage review process guided by Bandura's Self‐Efficacy Theory and principles of Person‐Centered Care. An initial pool of approximately 23 items was generated and organized into five preliminary domains: Knowledge and Skill Deficit, Care Philosophy and Approach, Intervention Preference, Helplessness and Pessimism, and Workload and Time Management.

The initial item pool was reviewed by an expert panel comprising six members, including four academic nursing researchers with expertise in dementia care and two clinical dietitians. Experts independently rated each item for relevance, clarity, and representativeness and provided qualitative feedback on wording, redundancy, and domain coverage. Based on this feedback, four items were removed due to redundancy, overlap across domains, or limited clarity. In addition, the panel identified a conceptual gap related to ethical tensions between resident autonomy and task efficiency, leading to the addition of one new item (Gauci et al. 2024).

To further assess face validity and cultural appropriateness, six registered nurses from an aged care setting not involved in the main study reviewed the revised scale. Minor wording and sequencing adjustments were made to enhance clarity and reduce ambiguity. The refined 19‐item instrument was subsequently re‐reviewed by four of the original experts, resulting in minor terminology refinements within the autonomy‐related items. No further items were added or removed at this stage. All revisions were discussed and finalized by consensus among the four co‐authors, with a revision log maintained.

This iterative process resulted in a refined 19‐item instrument with satisfactory content relevance and representativeness, suitable for subsequent psychometric evaluation (Yip et al. 2016).

#### 2.2.3 Theoretical Framework

The Assisting Mealtime Scale was informed by Bandura's Self‐Efficacy Theory and principles of person‐centered care (PCC). Self‐efficacy theory highlights individuals' beliefs in their capacity to manage complex tasks and challenging situations, which may influence how nursing staff perceive and respond to eating difficulties, behavioral resistance, and safety concerns in dementia care (Batchelor‐Murphy et al. 2015).

However, rather than directly measuring self‐efficacy, the scale captures a broader range of constructs relevant to mealtime support, including perceived barriers, task difficulty, emotional strain, and attitudinal responses. These elements can be understood as reflecting aspects of capability appraisal in clinical practice, without being limited to efficacy beliefs alone.

PCC principles informed items addressing resident autonomy, ethical decision‐making, and individualized mealtime support, ensuring that relational and contextual domains of care were incorporated (McCormack and McCance 2006).

Together, these frameworks provided a conceptual lens for item development and exploratory factor analysis, situating perceived knowledge gaps, workload pressures, and attitudinal beliefs within a broader understanding of caregiving experiences in person‐centered dementia care.

### 2.3 Phase 2: Scale Development and Initial Evaluation

#### 2.3.1 Pilot Testing and Cross‐Cultural Adaptation

Initial pilot testing was conducted with six nursing staff from an aged care setting not involved in the main study. Participants completed the draft instrument and provided structured feedback regarding clarity, wording, interpretability, and ease of completion. Minor refinements were made to item phrasing and sequencing to enhance readability and reduce ambiguity. No items were removed at this stage.

Given recruitment from both Australia and China, a structured forward–backward translation procedure was undertaken prior to administration in China to ensure semantic and conceptual equivalence across language versions. The original English instrument was independently translated into Chinese by two bilingual researchers with expertise in dementia nursing and aged care. The translations were reconciled through consensus discussion, prioritizing conceptual equivalence over literal wording. The reconciled Chinese version was subsequently back‐translated into English by an independent bilingual translator blinded to the original instrument. The back‐translation was systematically compared with the original English version to identify discrepancies, and minor refinements were introduced to ensure semantic consistency.

The Chinese version was then pilot tested with six registered nurses working in aged care settings in China to assess clarity, contextual relevance, and cultural appropriateness. Only minor wording adjustments were required, and no substantive conceptual modifications were identified.

As the primary aim of this study was exploratory instrument development rather than cross‐national comparison, data from Australia and China were pooled to enhance statistical power and stability for exploratory factor analysis. The combined sample (*N* = 240) yielded approximately 12.6 participants per item, exceeding recommended participant‐to‐item ratios of 5–10 for EFA and supporting factor extraction stability. Preliminary inspection suggested broadly comparable inter‐item correlation patterns across subsamples. However, formal multi‐group measurement invariance testing was not undertaken at this exploratory stage. Accordingly, pooled findings should be interpreted as identifying preliminary latent structure rather than establishing cross‐cultural equivalence.

#### 2.3.2 Participants and Data Collection

Sample size was determined using the recommended guideline of 5–10 participants per item, with an additional 20% allowance for incomplete responses (Shune and Linville 2019). From January to October 2024, nursing staff were recruited using purposive and convenience sampling from aged care settings in Sydney, Australia, and Chengdu, China.

Although participants were drawn from two countries, the study was not designed to compare nursing staff perceptions across cultural contexts. Instead, data were pooled to support exploratory factor identification during initial scale validation.

Eligible participants included registered nurses and nursing assistants with at least 2 weeks of experience assisting people living with dementia during mealtimes. Participants were required to be able to complete the consent the survey independently. Nursing staff employed in full‐time, part‐time, or temporary roles were eligible to participate.

Data were collected through an anonymous online self‐administered survey. Organizational consent was obtained from participating facilities, and trained researchers coordinated survey distribution. Participation was voluntary, and submission of the completed and submission of the questionnaires were taken as evidence of informed consent. A total of 240 complete questionnaires were received and included in the exploratory factor analysis.

#### 2.3.3 Sample Adequacy

Sampling adequacy was evaluated using the Kaiser–Meyer–Olkin (KMO) measure and Bartlett's test of sphericity. The overall KMO value was 0.891, indicating excellent sampling adequacy for factor analysis. Bartlett's test was statistically significant (*p* < 0.001), confirming that the correlation matrix was appropriate for factor analysis.

#### 2.3.4 Instrument

The final instrument comprised 19 items rated on a 5‐point Likert scale (1 = *strongly disagree* to 5 = *strongly agree*). Items were primarily negatively framed to capture perceived barriers, emotional strain, and contextual challenges associated with mealtime assistance. One item assessed willingness to assist residents despite time pressure.

Higher scores indicated greater perceived challenges and constraints in supporting people living with dementia during mealtimes.

#### 2.3.5 Data Analysis

Descriptive statistics were used to summarize participant characteristics and item distributions. Exploratory factor analysis (EFA) was conducted using Principal Axis Factoring (PAF). Given the conceptual interrelatedness of domains such as knowledge gaps, emotional strain, and organizational constraints, an oblique rotation method (Promax with Kaiser normalization) was applied to allow factors to correlate.

Factor retention was determined using multiple criteria, including eigenvalues greater than 1, inspection of the scree plot, and conceptual interpretability. A minimum of three items per factor was required to ensure structural stability.

Item retention criteria were specified a priori. Items were considered for retention if they demonstrated a primary factor loading ≥ 0.30 in the Promax‐rotated Pattern Matrix (Tavakol and Dennick 2011). Cross‐loadings were evaluated by comparing the magnitude of primary and secondary loadings. Although several items demonstrated modest cross‐loadings, primary loadings were generally stronger than secondary loadings, and items were retained based on overall interpretability and conceptual coherence within their respective domains. In cases where loading differences were modest, decisions were guided by theoretical alignment and the clarity of the overall factor structure. Extraction communalities ≥ 0.30 were preferred to indicate adequate shared variance with the retained factors.

Final retention decisions were informed by both statistical performance and theoretical alignment with the underlying construct domains. Internal consistency reliability was evaluated using Cronbach's alpha, with values ≥ 0.70 considered acceptable (A. Field 2013).

## 3 Results

The final version of the scale consisted of 19 items, each rated on a 5‐point Likert scale, and organized into three factors, which are referred to consistently throughout the manuscript.

Inter‐item correlations did not indicate redundancy (no correlations > 0.80), suggesting that conceptually similar items were not statistically duplicative.

### 3.1 Participants Characteristics

Table 1 summarizes the demographic characteristics of the participants. Data were collected between January and October 2024 from 240 nursing staff involved in mealtime assistance for older people in aged care settings in Australia (43.3%, *n* = 104) and China (56.7%, *n* = 136). Most participants were female (86%, *n* = 205), followed by males (13%, *n* = 32), with 1% (*n* = 3) preferring not to disclose their gender. Nearly half of the participants had a high school education or below (49.6%, *n* = 119), and 32.1% (*n* = 77) held a Certificate III or IV in aged care. Participants reported an average of approximately 4 years of experience working in aged care.

**TABLE 1:** Participant characteristics (*N* = 240).

Items	Minimum	Maximum	Means	*n*	%
Age	18	66	48 ± 10.133	240	—
Current aged care work experiences (years)	0	32	3.63 ± 3.693	240	—
Gender					
Female	—	—	—	205	86%
Male	—	—	—	32	13%
Unwilling to answer				3	1%
Country					
Australia				104	43.3%
China				136	56.7%
Education					
High school or below				119	49.6%
Certificate in aged care 3 or 4	—	—	—	77	32.1%
Diploma	—	—	—	12	5%
Bachelor	—	—	—	26	10.8%
Master	—	—	—	6	2.5%
Category of job					
Registered Nurse	—	—	—	135	56.3%
Enrolled Nurse	—	—	—	6	2.5%
Assistant in Nursing	—	—	—	95	39.9%
Other (social worker)	—	—	—	4	1.3%
Type of job					
Full‐time				145	60.4%
Part‐time				65	27.1%
Casual work				30	12.5%

### 3.2 Descriptive Statistics and Reliability Diagnostics

The 19‐item scale demonstrated good internal consistency (Cronbach's *α* = 0.890; standardized *α* = 0.895). Corrected item–total correlations ranged from 0.080 to 0.702, with the majority of items exceeding the recommended 0.30 threshold. Deletion of any single item did not meaningfully improve overall reliability, with the exception of Item 10, which demonstrated a low item–total correlation (0.080) and a marginal increase in alpha if removed (*α* = 0.897). Although Item 10 demonstrated a low corrected item–total correlation and extraction communality, it was retained due to its conceptual relevance to fatalistic beliefs regarding dementia progression. Given its low statistical performance, Item 10 warrants careful evaluation in future confirmatory modeling, including potential rewording or removal if it continues to demonstrate weak loading or low shared variance. All other items contributed adequately to scale reliability. (see Table 2).

**TABLE 2:** Item descriptive statistics and reliability diagnostics for the 19‐item scale (*N* = 240).

Items	*M*	SD	Corrected item–total correlation	α if item deleted
1	2.18	1.32	0.643	0.881
2	2.64	1.55	0.279	0.893
3	1.83	1.10	0.669	0.881
4	2.85	1.51	0.422	0.888
5	2.25	1.45	0.533	0.884
6	2.17	1.52	0.459	0.887
7	1.95	1.26	0.695	0.879
8	2.15	1.25	0.634	0.881
9	2.08	1.23	0.613	0.882
10	3.69	1.23	0.080	0.897
11	2.23	1.40	0.414	0.888
12	2.06	1.34	0.564	0.883
13	2.46	1.46	0.538	0.884
14	2.15	1.26	0.606	0.882
15	1.90	1.22	0.702	0.879
16	1.82	1.13	0.554	0.884
17	2.26	1.50	0.406	0.889
18	1.64	1.17	0.526	0.885
19	2.05	1.33	0.638	0.881

*Note:* Overall Cronbach's *α* = 0.890 (standardized α = 0.895). All items were scored on a 1–5 Likert scale. Higher scores indicate stronger endorsement of the construct.

### 3.3 Factorial Validity

Factorial validity was examined to assess the underlying structure of the instrument. The Kaiser–Meyer–Olkin (KMO) measure of sampling adequacy was 0.891, indicating excellent adequacy for factor analysis. Bartlett's test of sphericity was statistically significant (*p* < 0.001), confirming that the correlation matrix was not an identity matrix and that sufficient inter‐item correlations existed to justify factor extraction.

**FIGURE 1:**
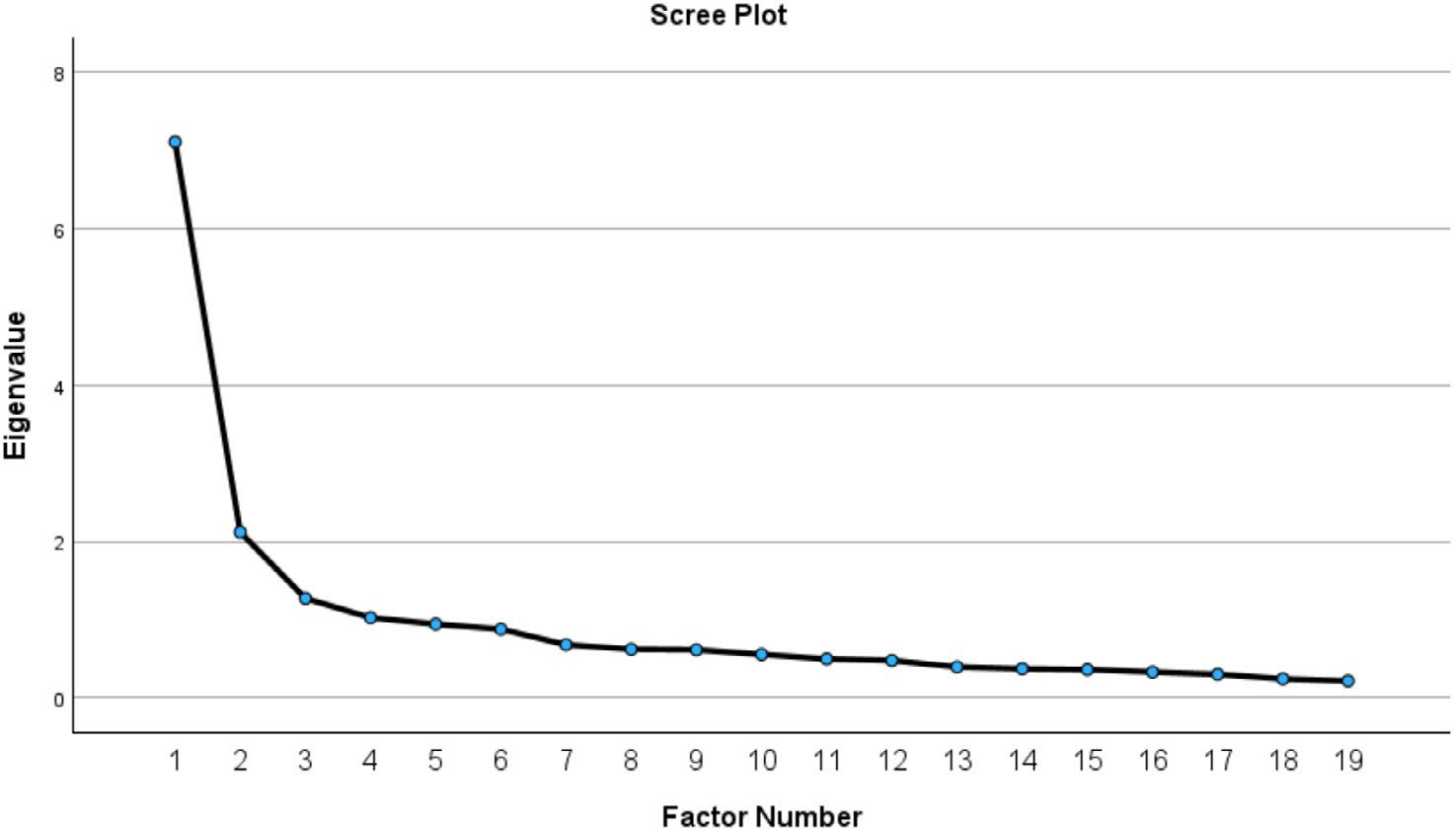
Scree plot indicating the number of extracted factors.

Exploratory factor analysis using PAF with Promax rotation yielded three interpretable factors. The first three factors had initial eigenvalues of 7.108, 2.117, and 1.271, collectively accounting for 55.25% of the total variance. Although a fourth factor had an eigenvalue slightly greater than 1 (1.025), visual inspection of the scree plot (Figure 1) revealed a clear inflection point after the third factor. Additionally, the three‐factor solution demonstrated conceptual coherence and interpretability, whereas the fourth factor did not represent a distinct or theoretically meaningful construct. Therefore, a three‐factor solution was retained.

The retained factors reflected conceptually coherent and interrelated domains of nursing staff perceptions and attitudes toward assisting people living with dementia during mealtimes. The Promax rotation revealed moderate inter‐factor correlations, indicating that while the domains are distinguishable, they are meaningfully associated. This pattern supports the appropriateness of an oblique rotation method and reinforces the multidimensional structure of the scale.

### 3.4 Item Communalities

PAF was conducted to examine the shared variance among the 19 items assessing staff perceptions and experiences related to assisting people living with dementia during mealtimes.

Initial communalities ranged from 0.252 to 0.680, indicating moderate shared variance among most items prior to extraction. After extraction, communalities ranged from 0.232 to 0.717, suggesting that the retained factor solution explained a reasonable proportion of variance for most items.

Most items demonstrated acceptable extraction communalities (≥ 0.30), indicating adequate shared variance with the retained factors. In particular, higher communalities were observed for:

- *Item 15* (“I do not know how to deal with people living with dementia refusal to eat”) (*h* ^2^ = 0.717)
- *Item 19* (“Assisting people living with dementia with food intake is the most difficult task…”) (*h* ^2^ = 0.660)
- *Item 12* (“Providing people living with dementia with eating takes too much time”) (*h* ^2^ = 0.643)
- *Item 7* (“I do not have confidence to help people living with dementia… eat safely via oral”) (*h* ^2^ = 0.607)

These findings indicate that the factor solution explains a substantial proportion of variance in items reflecting perceived difficulty, lack of confidence, and workload burden.

However, several items showed relatively low extraction communalities (< 0.30–0.35), including:

- *Item 10* (“Food intake difficulties of people living with dementia are inevitable…”) (*h* ^2^ = 0.232)
- *Item 2* (“Tube feeding is the best way…”) (*h* ^2^ = 0.261)
- *Item 4* (*h* ^2^ = 0.343)
- *Item 6* (*h* ^2^ = 0.344)

These findings indicate that certain items may be psychometrically weaker and may require revision or further evaluation in future confirmatory analyses. Overall, the communality pattern suggests that the retained factor solution accounts for an acceptable proportion of variance across most items in this exploratory analysis. However, the presence of several lower‐communality items should be taken into consideration when interpreting the results, and further validation using confirmatory factor analysis and cross‐cultural measurement invariance testing is warranted (see Table 3).

**TABLE 3:** Initial and extraction communalities for the assisting mealtime scale (principal axis factoring, *N* = 240).

Items	Initials	Extractions
1. I don't know how to deal with the eating difficulties of people living with dementia.	0.549	0.56
2. Tube feeding is the best way to solve the food intake difficulties of people living with dementia.	0.331	0.261
3. I cannot do anything when people living with dementia have food intake difficulties.	0.592	0.583
4. Taking care of people living with dementia with tube feeding is much easier than food intake via oral.	0.389	0.343
5. I do not have enough training to take care of people living with dementia with food intake difficulties.	0.468	0.478
6. In order to save working hours, I will assist a person living with dementia to eat even though he/she can eat by himself/herself.	0.355	0.344
7. I do not have confidence to help people living with dementia with food intake difficulties to eat safely and properly via oral.	0.61	0.607
8. Even effective eating assistance skills still cannot help people living with dementia with food intake difficulties.	0.532	0.45
9. No matter what I do, people living with dementia still get worse.	0.555	0.454
10. Food intake difficulties of people living with dementia are inevitable and can be worse.	0.252	0.232
11. I do not like to assist people living with dementia with eating.	0.418	0.397
12. Providing people living with dementia with eating takes too much time.	0.57	0.643
13. I am afraid of people living with dementia refusal to eat while assisting them.	0.438	0.396
14. I do not know how to deal with people living with dementia choking.	0.47	0.443
15. I do not know how to deal with people living with dementia refusal to eat.	0.68	0.717
16. I can aid with two or more people living with dementia at one time.	0.443	0.415
17. People living with dementia do not need to be given choices about what kinds of food to eat.	0.402	0.481
18. Assisting people living with dementia with eating should be done as fast as possible.	0.441	0.397
19. Assisting people living with dementia with food intake is the most difficult task of taking care of a person with dementia.	0.63	0.66

*Note:* Extraction method: principal axis factoring.

### 3.5 Three Factors Identified in Exploratory Factor Analysis (EFA)

Exploratory factor analysis using PAF with Promax rotation identified a three‐factor solution. Sampling adequacy was excellent (KMO = 0.891), and Bartlett's test of sphericity was statistically significant, *χ* ^2^(171) = 2038.811, *p* < 0.001, confirming the suitability of the data for factor analysis. Factor retention was guided by multiple criteria, including eigenvalues greater than 1, inspection of the scree plot, and conceptual interpretability. The retained three‐factor solution accounted for 55.25% of the total variance, and rotation converged after seven iterations.

The three factors were labeled “Perceived Knowledge and Skill Gaps”, “Time and Task Pressure”, and “Attitudes Toward Mealtime Assistance and Autonomy”. Cross‐loadings were generally modest; however, given the exploratory nature of the analysis, cross‐loading items were retained based on the strength of the primary loading in combination with conceptual coherence and should be interpreted with caution. Where items demonstrated more than one loading, final retention was based on the strength of the primary loading in combination with conceptual coherence. Inter‐factor correlations ranged from 0.221 to 0.653, indicating that the factors were related but distinguishable, although some degree of overlap was observed (see Table 4).

**TABLE 4:** Exploratory factor analysis of the assisting mealtime scale (principal axis factoring with promax rotation, *N* = 240).

Items	Factor 1 perceived knowledge and skill gaps	Factor 2 time and task pressure	Factor 3 attitudes toward mealtime assistance and autonomy
1. I do not know how to deal with eating difficulties.	**0.776**		
3. I cannot do anything when people living with dementia have food intake difficulties.	**0.736**		
5. I do not have enough training.	**0.774**		
7. I do not have confidence to assist safely.	**0.725**		
8. Even effective skills cannot help.	**0.448**		
9. No matter what I do, people living with dementia still get worse.	**0.590**		
14. I do not know how to deal with choking.	**0.619**		
15. I do not know how to deal with refusal to eat.	**0.847**		
12. Providing eating takes too much time.	**0.400**	**0.512**	
19. Assisting is the most difficult task.	**0.377**	**0.547**	
16. I can aid two or more people living with dementia at one time.	**0.361**	**0.364**	
6. I assist to save working hours.		**0.570**	
18. Assistance should be done as fast as possible.		**0.549**	
11. I do not like assisting with eating.	**0.399**		**0.462**
2. Tube feeding is the best solution.			**0.490**
4. Tube feeding is easier than oral feeding.			**0.494**
10. Difficulties are inevitable and worsen.			**0.481**
13. I am afraid of refusal during assistance.			**0.408**
17. People living with dementia do not need food choices.			**0.604**

*Note:* Factors were extracted using Principal Axis Factoring and rotated using Promax with Kaiser normalization. Only loadings ≥ 0.30 are shown, with smaller loadings suppressed for clarity. Bolded values indicate the primary loading on which each item was assigned to its final retained factor. Cross‐loadings are presented for transparency but were not used for multiple assignment in scoring. The final factor structure comprised Factor 1 (Items 1, 3, 5, 7, 8, 9, 14, and 15), Factor 2 (Items 6, 12, 16, 18, and 19), and Factor 3 (Items 2, 4, 10, 11, 13, and 17).

*Factor 1: Perceived Knowledge and Skill Gaps* included items reflecting perceived lack of knowledge, training, and confidence in managing mealtime difficulties among people living with dementia. Primary loadings ranged from 0.361 to 0.847, with the strongest loadings observed for difficulty managing refusal to eat (0.847), lack of knowledge in handling eating difficulties (0.776), insufficient training (0.774), inability to manage food intake difficulties (0.736), and lack of confidence in assisting oral feeding safely (0.725).

*Factor 2: Time and Task Pressure* comprised items reflecting workload burden and time‐related pressures during mealtime assistance. Primary loadings ranged from 0.364 to 0.570. Items in this factor captured themes such as assisting residents to save time (0.570), completing feeding tasks quickly (0.549), perceiving mealtime assistance as the most difficult caregiving task (0.547), and the time burden associated with feeding support (0.512).

*Factor 3: Attitudes Toward Mealtime Assistance and Autonomy* included items reflecting beliefs about tube mealtime assistance, nutritional decline, and resident choice during mealtimes. Primary loadings ranged from 0.408 to 0.604. The strongest loadings included the view that residents do not need food choices (0.604), the perception that tube feeding is easier than oral feeding (0.494), endorsement of tube feeding as the best solution for intake difficulties (0.490), and the belief that feeding decline is inevitable (0.481).

The Promax‐rotated factor correlation matrix showed a moderate association between Factor 1 and Factor 2 (*r* = 0.653), and weaker associations between Factors 1 and 3 (*r* = 0.221) and Factors 2 and 3 (*r* = 0.269), indicating that the three domains were conceptually related but distinct. However, these correlations also suggest some shared variance between factors, which should be considered when interpreting the dimensionality of the scale.

The overall 19‐item scale demonstrated good internal consistency (Cronbach's *α* = 0.890). Subscale reliability was excellent for Perceived Knowledge and Skill Gaps (*α* = 0.904), acceptable for Time and Task Pressure (*α* = 0.790), and acceptable for Attitudes Toward Mealtime Assistance and Autonomy (*α* = 0.712). It should be noted that these reliability estimates should be interpreted as preliminary evidence within the context of an exploratory study, pending further validation.

## 4 Discussion

The primary objective of this study was to develop and conduct an exploratory evaluation of a scale designed to assess nursing staff perspectives and experiences in supporting older people living with dementia during mealtimes. Exploratory factor analysis identified a theoretically coherent three‐factor structure accounting for 55.25% of total variance, providing preliminary evidence supporting the multidimensional nature of the construct. The retained factors—Perceived Knowledge and Skill Gaps, Time and Task Pressure, and Attitudes Toward mealtime assistance and Autonomy—represent interrelated yet distinguishable domains of mealtime care challenges. The proportion of explained variance is comparable to that reported in other exploratory instrument development studies within nursing and dementia care research (A. Field 2013; Tavakol and Dennick 2011), although such comparisons should be interpreted cautiously given differences in study design and sample characteristics. Overall, these findings offer initial support for the internal structure of the instrument, rather than definitive validation. Collectively, these findings indicate that dementia mealtime care challenges arise from the interaction between individual capability appraisals and organizational constraints, reinforcing the need for multilevel workforce interventions.

A total of 240 particpants completed questionnaires were received, with completation and submission taken as evidence of informed consent, reflecting strong engagement and underscoring the clinical relevance of mealtime care in everyday practice. Similar engagement has been reported in studies examining feeding difficulties, nutritional care, and frontline nursing experiences in residential aged care, suggesting that mealtime assistance is widely recognized as both challenging and essential in dementia care (Training.gov.au 2024; Robinson 2014; Chenoweth 2010; Saucedo Figueredo et al. 2023). This high participation rate supports the feasibility and practical relevance of the study; however, it should not be interpreted as evidence of representativeness beyond the study sample.

Interpretation of the results must be situated within the intent of the study. Although many items were negatively phrased, the scale is not intended to portray nursing staff attitudes as inherently negative or stigmatizing. Rather, the items capture perceived barriers, emotional strain, and organizational challenges encountered during mealtime assistance for people living with dementia. This framing aligns with previous research that conceptualizes staff responses as reflections of systemic and contextual constraints rather than individual shortcomings (Chang et al. 2023; Chen et al. 2023). Such an approach supports ethical interpretation and constructive application for workforce development and care improvement.

### 4.1 Demographic Insights and Workforce Characteristics

The demographic profile of participants broadly reflects patterns reported in aged care research, including a predominantly female workforce and a midlife average age (Training.gov.au 2024; Robinson 2014). The high proportion of staff holding Certificate III or IV qualifications is consistent with vocational training pathways typical of frontline aged care roles (Chenoweth 2010). Participants reported a mean of approximately 4 years of aged care experience; however, experience alone does not necessarily equate to preparedness to manage complex eating difficulties (Chang et al. 2023; Perry 2011). These characteristics provide contextual grounding for the interpretation of the scale findings, particularly the prominence of Perceived Knowledge and Skill Gaps, Time and Task Pressure. The results reinforce the need for structured professional development in dysphagia management, person‐centered feeding strategies, and ethical decision‐making during mealtimes.

### 4.2 Factorial Validity and Instrument Development

The methodological approach used in this study aligns with established guidelines for exploratory instrument development in nursing research. Face and content validity were strengthened through expert review and iterative refinement (A. Field 2013; Tavakol and Dennick 2011). The strong Kaiser–Meyer–Olkin value (0.891) and significant Bartlett's test of sphericity confirmed the adequacy of the sample for factor analysis.

The three‐factor structure highlights the complexity of nursing staff experiences during dementia mealtime care. The Promax rotation revealed moderate correlations between Perceived Knowledge and Skill Gaps and Time and Task Pressure (*r* = 0.653), and weaker correlations involving Attitudes Toward Mealtime Assistance and Autonomy (*r* = 0.221–0.269). These findings support the appropriateness of oblique rotation and suggest that the domains represent related but distinguishable aspects of nursing staff perceptions. However, the observed correlations also indicate some degree of overlap among domains, which should be considered when interpreting the factor structure. The moderate association between Perceived Knowledge and Skill Gaps and Time and Task Pressure suggests that perceived knowledge and skill gaps limitations may be compounded by organizational constraints. The relatively large number of items loading onto Factor 1 further suggests that perceived capability‐related concerns represent a central dimension of mealtime care challenges rather than a peripheral construct. This pattern may reflect the prominence of capability‐related perceptions within the sample, although it should not be interpreted as definitive evidence of construct hierarchy.

This concentration of items may reflect the centrality of perceived capability appraisal within the theoretical framework underpinning the instrument, rather than a statistical artifact of extraction. The prominence of the capability‐related factor is theoretically consistent with Bandura's conceptualization of self‐efficacy as a central determinant of behavioral engagement in complex care situations (Faraday et al. 2019), although the present findings provide only indirect and preliminary support for this interpretation.

### 4.3 Interpretation of Identified Factors in Relation to Existing Literature

The three‐factor structure suggests that nursing staff perspectives on dementia mealtime care are shaped by capability‐related concerns, organizational pressures, and attitudinal beliefs. Perceived Knowledge and Skill Gaps indicate that staff may feel insufficiently prepared to manage eating difficulties in people living with dementia, which is consistent with previous research describing limited training in dysphagia management, behavioral eating challenges, and safe oral feeding techniques (Batchelor‐Murphy et al. 2015; Chang et al. 2023; Chang, Lin, et al. 2017). Such gaps in preparation may contribute to uncertainty and greater reliance on task‐focused rather than individualized mealtime support.

Time and Task Pressure highlight the influence of workload burden and organizational constraints on mealtime care. This finding aligns with existing literature showing that time pressure and staffing limitations are important barriers to person‐centered dementia care, particularly during mealtimes (Li et al. 2021a). Organizational priorities that emphasize efficiency may reduce opportunities for relational and individualized support.

Attitudes Toward Mealtime Assistance and Autonomy reflects beliefs about tube eating, inevitability of decline, and resident choice during meals. The weaker correlations between this factor and the other domains suggest that these attitudinal beliefs may represent a somewhat more distinct dimension of mealtime care. Ethical tensions surrounding autonomy, safety, and nutritional adequacy have been widely discussed in the dementia care literature (Batchelor‐Murphy et al. 2015), and the present findings highlight the importance of addressing both practical and attitudinal domains in staff education and organizational policy.

Taken together, these findings suggest rather than confirm that challenges in dementia mealtime care are shaped not only by staff preparedness, but also by organizational and belief‐based influences within care environments. This pattern supports the need for multilevel strategies that strengthen staff capability, reduce structural pressures, and promote person‐centered approaches to mealtime support.

## 5 Study Strength and Limitation

This study has several strengths. It employed a systematic, multi‐phase instrument development process, including literature review, multidisciplinary expert consultation, pilot testing, and exploratory psychometric evaluation. This structured approach enhanced content validity, conceptual clarity, and clinical relevance. The use of exploratory factor analysis with oblique rotation enabled identification of related yet distinguishable domains of nursing staff perceptions, while internal consistency testing provided preliminary evidence of reliability (A. Field 2013). Together, these procedures support the methodological transparency and exploratory rigor of the instrument development process.

Importantly, the scale captures three interrelated domains of dementia mealtime care: perceived knowledge and skill gaps, time and task pressure, and attitudinal beliefs related to mealtime assistance and autonomy. By reflecting both individual perceptions and organizational influences, the instrument recognizes that mealtime challenges arise from the interaction between staff preparedness, emotional demands, and structural constraints. Inclusion of nursing staff from two care systems further enhances the potential contextual relevance of the scale across varied practice contexts, although generalizability beyond the study sample remains limited.

Several limitations should be acknowledged. As an exploratory validation study, confirmatory factor analysis, convergent validity testing, and differential item functioning were not conducted. The absence of parallel analysis represents a methodological limitation. In addition, pooling data across two countries without formal multi‐group testing limits the ability to access cross‐cultural equivalence and may obscure potential differences in factor structure. Although multiple retention criteria were applied to support factor selection, future validation should incorporate parallel analysis and confirmatory factor analysis to enhance structural robustness. The three‐factor structure therefore requires replication in independent samples.

The cross‐sectional design limits causal interpretation, and reliance on self‐reported data may introduce response bias. Although translation procedures supported semantic equivalence, formal cross‐cultural measurement invariance testing was not performed. Future research should examine structural stability and invariance across cultural settings before broader implementation, and should further evaluate item performance, particularly for items that demonstrated weaker psychometric properties in the present study.

## 6 Implications for Healthcare Practice and Future Research

This study advances understanding of nursing staff perspectives and challenges in supporting people with dementia during mealtimes and provides practical insights to inform targeted interventions aimed at improving nutritional care and overall dementia care quality. Managing the health and nutrition of people with dementia is complex, underscoring the need for validated or well‐developed instruments that may help identify staff support needs and guide personalized education and assistance (Gaskin and Happell 2014).

The 19‐item Assisting Mealtime Scale may provide a structured approach for assessing nursing staff challenges related to dementia mealtime care. By capturing key barriers and support needs, the scale has potential to assist in identifying areas requiring further attention, although its application should be considered preliminary and subject to further validation in different contexts.

Based on the findings, several actionable strategies emerge. These include strengthening training programs to enhance practical feeding skills and confidence in managing eating difficulties (Batchelor‐Murphy et al. 2015; Chang et al. 2023; Chang, Lin, et al. 2017; Chen et al. 2023), implementing emotional support mechanisms such as peer support or counseling to address psychological strain (Perry 2011), optimizing workflows and staffing to reduce time pressures, and promoting person‐centered care approaches that respect the autonomy and food preferences of people with dementia (Kigozi et al. 2021). Together, these strategies may contribute to improvements in care quality and nutritional outcomes.

Future research should examine the stability of the identified factor structure and evaluate the effectiveness of targeted workforce development strategies over time. Comparative studies across diverse care settings may further refine context‐specific strategies to support nursing staff and enhance dementia mealtime care (Li et al. 2021a). In addition, further psychometric evaluation of the scale, including confirmatory factor analysis, measurement invariance testing, and item refinement, is warranted before broader implementation.

## 7 Conclusion

This study reports the initial development and exploratory validation of the Assisting Mealtime Scale, a 19‐item instrument designed to assess nursing staff perspectives on supporting people living with dementia during mealtimes. Exploratory factor analysis identified a three‐factor structure comprising perceived knowledge and skill gaps, time and task pressure, and attitudes toward mealtime assistance and autonomy. These domains reflect key perceived barriers, organizational constraints, and attitudinal influences shaping mealtime care practices. The overall scale demonstrated acceptable internal consistency, providing preliminary evidence of reliability and internal structural coherence.

The findings suggest that challenges in dementia mealtime care are multifaceted and influenced by both individual preparedness and structural conditions within care environments. The Assisting Mealtime Scale may provide a useful exploratory framework for examining these interrelated domains and has potential to assist in identifying areas for training and workforce support in research and practice contexts, although its application should remain cautious and contingent upon further validation.

As an exploratory validation study based on pooled cross‐national data, the findings should be interpreted with caution. Further psychometric evaluation using independent samples is needed, including confirmatory factor analysis, assessment of convergent validity, and examination of measurement invariance across cultural and organizational contexts. In addition, refinement of items with weaker psychometric performance may further strengthen the scale. At this stage, the instrument should be considered as an exploratory tool, and not yet a fully validated measure, pending additional empirical evidence. With continued validation, the scale may contribute to future research aimed at better understanding and improving mealtime care for people living with dementia.

## 8 Relevance for Clinical Practice

The Assisting Mealtime Scale provides a structured approach to identifying nursing staff support needs related to dementia mealtime care. By highlighting perceived knowledge gaps, emotional strain, organizational constraints, and autonomy‐related challenges, the scale may inform targeted staff education, workforce support, and quality improvement initiatives aimed at enhancing mealtime experiences and nutritional care for people with dementia.

## Author Contributions

**Hansen Tang (Cindy):** conceptualization, vaildation, writing – original draft. **WenPeng You:** validation, formal analysis, reviewing and editing. **Kazem Razaghi:** writing – review and editing. **Fung Kuen (Tebbin) Koo:** writing – review and editing. **Hui‐Chen (Rita) Chang:** conceptualization, validation, writing – review and editing.

## Funding

The authors have nothing to report.

## Ethics Statement

This study followed established protocols and procedures for research involving human subjects. The study was approved by the Human Research Ethics Committees of Western Sydney University (H15406).

## Conflicts of Interest

Wenpeng You is an Associate Editor for Nursing and Health Sciences and a co‐author on this article. The manuscript was managed by editors unaffiliated with the author or institution and monitored carefully to ensure there is no peer review bias.

## Data Availability

The data that support the findings of this study are available on request from the corresponding author. The data are not publicly available due to privacy or ethical restrictions.
